# Efficient and cost-effective bacterial mRNA sequencing from low input samples through ribosomal RNA depletion

**DOI:** 10.1186/s12864-020-07134-4

**Published:** 2020-10-16

**Authors:** Chatarin Wangsanuwat, Kellie A. Heom, Estella Liu, Michelle A. O’Malley, Siddharth S. Dey

**Affiliations:** 1 Department of Chemical Engineering, University of California Santa Barbara, Santa Barbara, CA 93106 USA; 2 Center for Bioengineering, University of California Santa Barbara, Santa Barbara, CA 93106 USA; 3 Neuroscience Research Institute, University of California Santa Barbara, Santa Barbara, CA 93106 USA

**Keywords:** Bacterial mRNA sequencing, mRNA enrichment, rRNA depletion, Low input total RNA

## Abstract

**Background:**

RNA sequencing is a powerful approach to quantify the genome-wide distribution of mRNA molecules in a population to gain deeper understanding of cellular functions and phenotypes. However, unlike eukaryotic cells, mRNA sequencing of bacterial samples is more challenging due to the absence of a poly-A tail that typically enables efficient capture and enrichment of mRNA from the abundant rRNA molecules in a cell. Moreover, bacterial cells frequently contain 100-fold lower quantities of RNA compared to mammalian cells, which further complicates mRNA sequencing from non-cultivable and non-model bacterial species. To overcome these limitations, we report EMBR-seq (Enrichment of mRNA by Blocked rRNA), a method that efficiently depletes 5S, 16S and 23S rRNA using blocking primers to prevent their amplification.

**Results:**

EMBR-seq results in 90% of the sequenced RNA molecules from an *E. coli* culture deriving from mRNA. We demonstrate that this increased efficiency provides a deeper view of the transcriptome without introducing technical amplification-induced biases. Moreover, compared to recent methods that employ a large array of oligonucleotides to deplete rRNA, EMBR-seq uses a single or a few oligonucleotides per rRNA, thereby making this new technology significantly more cost-effective, especially when applied to varied bacterial species. Finally, compared to existing commercial kits for bacterial rRNA depletion, we show that EMBR-seq can be used to successfully quantify the transcriptome from more than 500-fold lower starting total RNA.

**Conclusions:**

EMBR-seq provides an efficient and cost-effective approach to quantify global gene expression profiles from low input bacterial samples.

## Background

Bacterial species pervade our biosphere and millions of years of evolution have optimized these microbes to perform specific biochemical reactions and functions; processes that could potentially be adapted to develop a variety of products, such as renewable biofuels, antibiotics, and other value-added chemicals [1–5]. Bacterial messenger RNA (mRNA) sequencing provides a snapshot of the genome-wide state of a microbial population, and therefore enables fundamental understanding of these varied microbial functions and phenotypes [6].

However, compared to eukaryotes, mRNA sequencing from bacterial samples has been more challenging for several reasons. First, unlike in eukaryotes, bacterial mRNA does not contain a poly-A tail at the 3′ end that can be used to easily enrich for these molecules during reverse transcription [7, 8]. Further, total RNA isolated from bacterial cells typically contains greater than 95% ribosomal RNA (rRNA), and therefore cost-effective and high coverage sequencing of the transcriptome requires the development of efficient strategies to deplete the abundant 5S, 16S and 23S rRNA molecules [9]. Finally, bacterial cells typically contain approximately 100-fold lower RNA than mammalian cells, and as the starting amount of total RNA when working with rare, non-cultivable, and non-model bacterial species can be limiting, it is a challenge to robustly and accurately capture the transcriptome from small quantities of total RNA with minimal amplification biases [10].

Several commercial kits have been developed to deplete bacterial rRNA from total RNA samples, including the MICROBExpress Bacterial mRNA Enrichment Kit (Thermo Fisher Scientific), the RiboMinus Transcriptome Isolation Kit, bacteria (Thermo Fisher Scientific), and the Ribo-Zero rRNA Depletion Kit (Illumina) [11]. These techniques rely on subtractive hybridization to deplete rRNA and typically work at a scale of hundreds of nanograms to micrograms of starting total RNA. Further, as these commercial kits are only effective on species targeted in the standard probe set, it is challenging to extrapolate these methods to diverse bacterial species [9, 11]. While this limitation of pre-designed kits has been overcome through the development of workflows to generate custom subtractive hybridization probe sets for any species of interest, they still operate at microgram quantities of starting material and either require multiple rounds of hybridization or a series of oligo optimization steps prior to optimal performance [12, 13]. An alternate approach relies on the Terminator™ 5′-phosphate-dependent exonuclease (TEX) (Lucigen) to specifically degrade rRNAs with 5′-monophosphate ends but not mRNAs with 5′-triphosphate ends; however, this method typically has lower efficiencies than other existing rRNA depletion strategies [10, 14, 15]. A more recent method uses complementary single-stranded DNA probes to tile rRNAs that are subsequently degraded by RNase H [16]. The commercial NEBNext Bacteria rRNA depletion kit (NEB) employs a similar strategy and can be applied to as low as 10 ng of starting total RNA. Similarly, another approach uses a pool of tiled single-guide RNAs to direct Cas9 mediated cleavage of rRNA-derived cDNA to deplete rRNA while another approach uses targeted reverse transcription primers designed to avoid capturing rRNAs [17, 18]. However, all these methods require a large array of probes that can be expensive to synthesize and potentially need to be redesigned for distant bacterial species [16–18].

Therefore, in this work we have developed EMBR-seq (Enrichment of mRNA by Blocked rRNA), a new technology that overcomes the limitations of sequencing mRNA from bacterial samples by: (1) Using 5S, 16S and 23S rRNA blocking primers and poly-A tailing to specifically deplete rRNA and enrich mRNA during downstream amplification; (2) Using a single or a few blocking primers for each of the three abundant rRNA molecules, thereby enabling rapid adaptation to different bacterial species and significantly reducing the cost per sample; and (3) Using a linear amplification strategy to amplify mRNA from as low as 20 picograms of total RNA with minimal amplification biases. We applied EMBR-seq to a model *E. coli* system to demonstrate efficient mRNA enrichment and sequencing with increased sensitivity in gene detection. Further, we show that our method accurately captures the genome-wide gene expression profiles with minimal technical biases. Thus, EMBR-seq is an efficient and cost-effective approach to sequence mRNA from low-input bacterial samples.

## Results

### EMBR-seq uses blocking primers to deplete rRNA

**Fig. 1:**
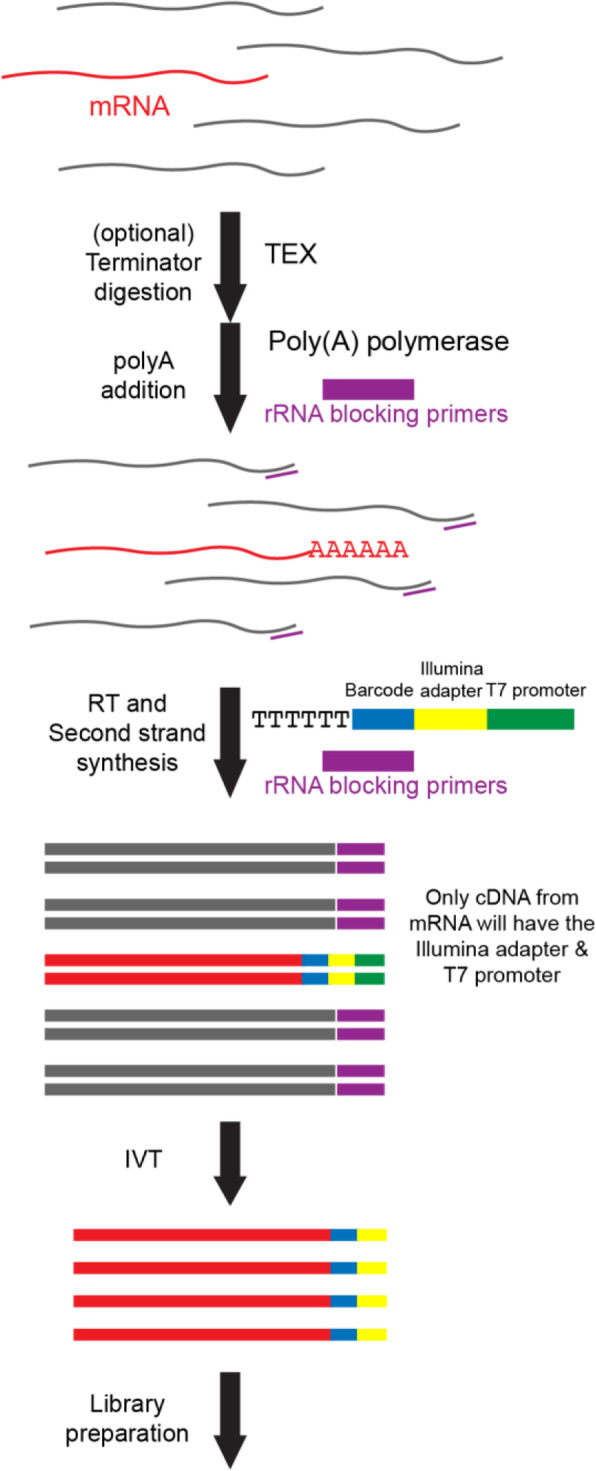
Schematic of EMBR-Seq. After performing an optional Terminator™ 5′-phosphate-dependent exonuclease digestion, poly-A polymerase and rRNA blocking primers (purple) are added to total bacterial RNA (mRNA in red and rRNA in gray). Blocking primers specifically bind to the 3′ end of 5S, 16S, and 23S rRNAs, resulting in the preferential addition of a poly-A tail to mRNA molecules. Next, reverse transcription is performed using (i) a poly-T primer, which has an overhang containing a sample-specific barcode (blue), 5′ Illumina adapter (yellow), and T7 promoter (green), and (ii) rRNA blocking primers to convert poly-adenylated RNA and rRNA molecules, respectively, to cDNA. The cDNA molecules are then amplified by in vitro transcription, and the amplified RNA is used to prepare Illumina libraries. As the rRNA-derived cDNA does not contain a T7 promoter, these molecules are not amplified during in vitro transcription, resulting in rRNA depletion

To overcome the limitations described above, we developed EMBR-seq, a new technique to efficiently deplete rRNA from total RNA, thereby enabling cost-effective sequencing of mRNA from bacterial cells. To minimize rRNA-derived molecules in the final sequencing library, we first incubated the total RNA with rRNA blocking primers, designed specifically to bind the 3′ end of 5S, 16S and 23S rRNA, followed by poly-adenylation with *E. coli* poly-A polymerase (Fig. 1 and Methods). To deplete rRNA, EMBR-seq only requires primers at the 3′ end of rRNA, unlike recent methods that tile oligonucleotides along the entire length of rRNA molecules, thereby significantly reducing costs and making our approach more easily translatable to other bacterial species. The blocking primers generate double-stranded RNA-DNA hybrid molecules at the 3′ end of rRNAs, which reduces subsequent poly-adenylation and downstream amplification of rRNA molecules, as the poly-A polymerase preferentially adds adenines to single-stranded RNA [19]. Thereafter, the reaction mixture is reverse transcribed following the addition of a poly-T primer. This primer has an overhang containing a sample-specific barcode to enable rapid multiplexing and reduction in library preparation costs, the 5′ Illumina adapter, and a T7 promoter [20]. After second strand synthesis, cDNA molecules are amplified by in vitro transcription (IVT). However, as only cDNA molecules deriving from a poly-adenylated RNA have a T7 promoter, our technique further amplifies mRNA-derived molecules for sequencing whereas rRNA-derived molecules are excluded from IVT amplification. The amplified RNA from IVT is then used to prepare Illumina sequencing libraries, as described previously (Fig. 1 and Methods) [20–22].

### EMBR-seq efficiently depletes rRNA to sequence bacterial mRNA

**Fig. 2:**
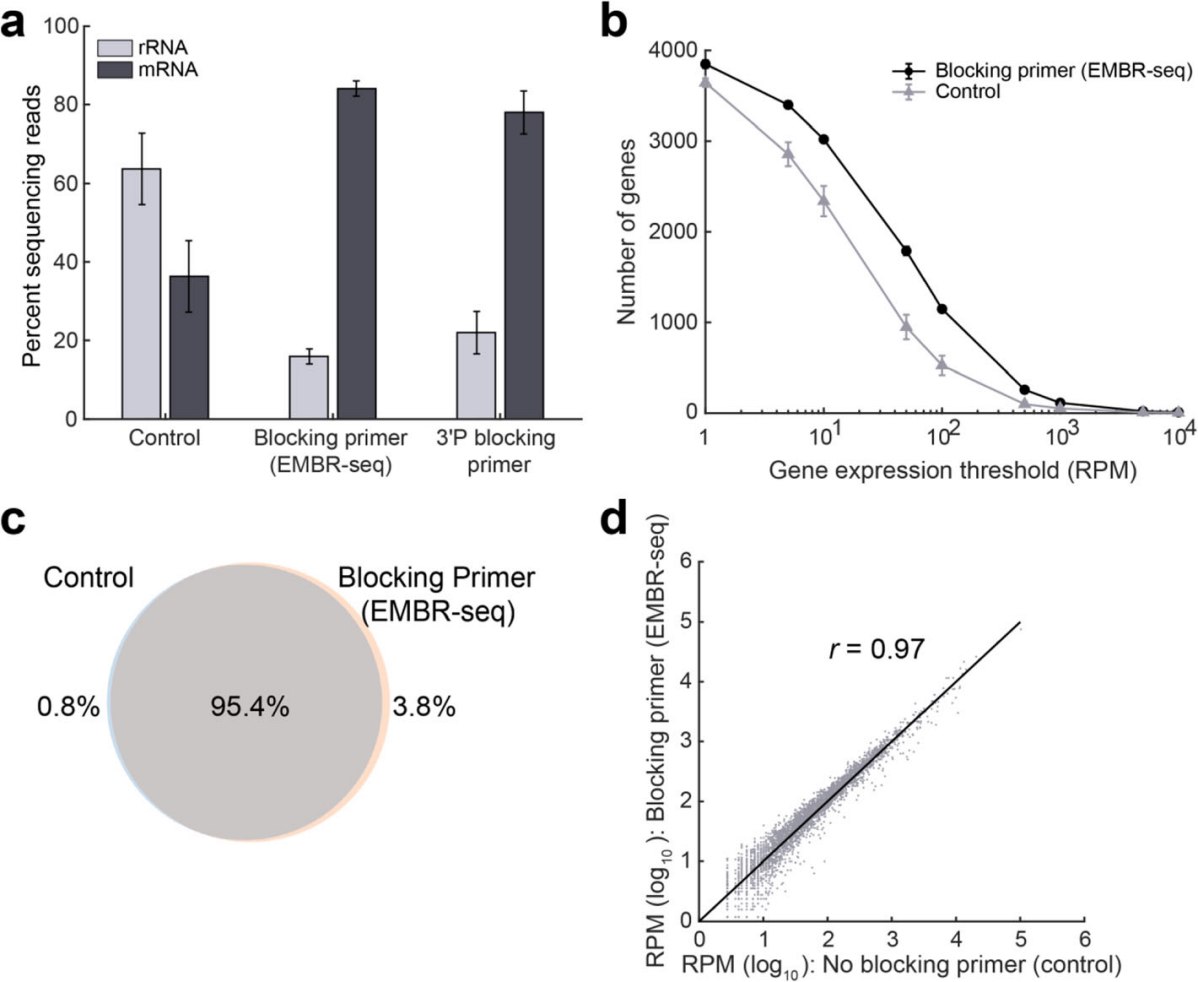
Blocking primers in EMBR-seq deplete rRNA and provide a deeper view of the transcriptome without introducing technical biases. **a** In the presence of blocking primers, a 4-fold rRNA depletion and more than 2-fold mRNA enrichment is achieved compared to control samples. With the introduction of blocking primers in EMBR-seq, mRNAs account for more than 80% of the mapped reads, which is a greater than 16-fold increase compared to total RNA in *E. coli* cells. The 3′ phosphorylated blocking primers display similar but slightly lesser mRNA enrichment (*n* ≥ 2 replicates for all conditions). **b** Comparison between EMBR-seq and control samples in the number of genes detected above different expression thresholds (*n* = 3 for both conditions). For the EMBR-seq group, error bars are of the same scale as the size of the data points. **c** Venn diagram shows that more than 99% of the genes detected in the control samples were also detected when using blocking primers in EMBR-seq. 99.2% of all detected genes were found in the EMBR-seq samples and 96.2% in the control samples. The number of genes detected were calculated by combining data obtained from three control samples and three EMBR-seq processed samples. **d** Gene transcript counts with and without blocking primers are highly correlated (Pearson *r* = 0.97) suggesting that EMBR-seq does not introduce technical artifacts in quantifying gene expression (*n* = 3 for both datasets). These experiments were performed starting with 100 ng total RNA from *E. coli.* Error bars in panels (**a**) and (**b**) represent standard deviations

We applied EMBR-seq to total RNA isolated from the exponential growth phase of *E. coli* strain K12 (MG1655). Starting from 100 ng of total RNA, we were able to successfully make Illumina libraries that were sequenced and mapped to the *E. coli* transcriptome. In parallel, we prepared control libraries where total RNA was processed using the EMBR-seq protocol but in the absence of blocking primers. While total RNA from *E. coli* has previously been reported to consist of 95% rRNA [9], our control samples with no blocking primers had approximately 64% rRNA, consistent with previous observations that mRNA molecules are preferentially poly-adenylated compared to rRNA even in the absence of any blocking primers (Fig. 2a) [23, 24]. Importantly, compared to the control samples, we observed a significant increase in rRNA depletion efficiency (from 64 to 16%), with 84% of the mapped reads corresponding to mRNA in samples treated with blocking primers (Fig. 2a). As tRNAs make up another major class of RNA molecules, we analyzed our data to quantify the detection of these molecules [25]. We found tRNA-derived reads to constitute only 0.37 and 1.26% of the mapped reads in the control and EMBR-seq samples, respectively. This expected low detection rate is likely due to the small size of tRNAs, their stable secondary structures and utilization of numerous post-transcriptionally modified nucleotides that are known to interfere with reverse transcription [26, 27]. Overall, these results demonstrate that EMBR-seq achieves a level of mRNA enrichment that is better or comparable to recent bacterial rRNA depletion reports [11–13, 15–18].

In certain applications, such as those where RNA is extracted from non-cultivable bacterial species within natural isolates, the total RNA can be fragmented and of poor quality. To determine if EMBR-seq can still be successfully applied to degraded RNA, we compared the rRNA depletion efficiency of total *E. coli* RNA with two different RIN (RNA Integrity Number) scores of 7.2 and 2.4. While RNA molecules can be fragmented by several mechanisms, the degraded sample with a RIN score of 2.4 in this experiment was prepared by heating the total RNA at 95 °C for 5 min. Surprisingly, we observed that EMBR-seq depleted rRNA to similar levels of 15 and 17% in the untreated and degraded samples, respectively (Additional file 1: Fig. S1). We hypothesize this occurs because both rRNA and mRNA molecules are fragmented to similar extents, and the increased poly-adenylation of rRNA fragments is matched by a similar increase in poly-adenylation of mRNA fragments, resulting in similar downstream detection of rRNA- and mRNA-derived reads. Thus, these results suggest that EMBR-seq can be effectively applied to sequence the transcriptome of randomly fragmented lower quality total RNA.

We also tested modified blocking primers with a 3′ phosphorylation, designed to prevent Superscript II from reverse transcribing rRNA molecules. As expected, we observed rRNA depletion in these samples as well (from 64 to 22%), with 78% of the mapped reads corresponding to mRNA (Fig. 2a). However, compared to the unmodified blocking primers, these phosphorylated blocking primers were slightly less efficient at rRNA depletion (Fig. 2a). As the 3′ phosphorylated primers prevent polymerase extension, we hypothesize that the reduced rRNA depletion efficiency arises from the small fraction of rRNA molecules that get poly-adenylated, primed by the poly-T primers, and copied through the short 30 bp RNA-DNA hybrid due to the strand-displacement activity of the reverse transcriptase. Therefore, given the reduced efficiency and higher costs of the 3′ phosphorylated blocking primers, all further experiments were performed with unmodified blocking primers.

As an alternate strategy, we also incorporated TEX treatment in EMBR-seq as it has previously been shown to specifically degrade rRNAs with 5′-monophosphate ends but not mRNAs that have 5′-triphosphate ends [10, 14, 15, 28]. While we again observed rRNA depletion and a corresponding enrichment of mRNA compared to control samples, the effects were less pronounced with a less than 2-fold rRNA depletion, consistent with previous reports (Additional file 1: Fig. S2) [14, 15]. We hypothesize that this reduced efficiency may arise from the additional cleanup step that is necessary prior to treatment with the poly-A polymerase. As a result, we find that blocking primers alone provide the most significant rRNA depletion and mRNA enrichment, and therefore all further experiments were performed without TEX treatment.

### EMBR-seq is a cost-effective bacterial mRNA sequencing technology

In designing the steps of EMBR-seq, we wanted to develop a method that is both easily applied and cost-effective. Due to its simplicity, the cost per rRNA depletion reaction in EMBR-seq is ~$0.40, which is at least an order of magnitude lower than other recent rRNA depletion methods and commercial kits [11–13, 15–18] (Additional file 1: Fig. S3a, Table S1, and Additional file 2). The total cost of EMBR-seq, starting from total bacterial RNA to the final Illumina library, was estimated to be ~$36 per sample. However, the total cost per sample decreases as more samples are multiplexed in the same Illumina library. For example, when 96 samples are multiplexed, the cost per sample drops to ~$20, primarily due to the pooling of samples after second-strand synthesis that then requires only a single IVT and Illumina library preparation reaction downstream (Additional file 1: Fig. S3b and Additional file 2). Thus, EMBR-seq is a simple and cost-effective approach to sequence mRNA from total bacterial RNA.

### EMBR-seq provides a detailed view of the transcriptome without introducing technical biases

Next, we systematically compared the gene expression profiles obtained from control and rRNA depleted samples to investigate if the use of blocking primers provides a deeper view of the transcriptome without introducing technical artifacts. First, after downsampling sequencing reads to the same depth, we detected 3628 genes in the control samples, while in the mRNA enriched samples we detected 3852 genes, with 99% of the genes in the control samples also detected in the mRNA enriched samples (Fig. 2b, c). Moreover, at different levels of downsampling, we detected more genes using EMBR-seq compared to the control samples (Additional file 1: Fig. S4). This suggests that we can measure the genome-wide gene expression landscape in a more cost-effective way using EMBR-seq. Further, the number of genes detected above different expression thresholds was consistently higher for the mRNA enriched samples compared to the control samples (Fig. 2b). This shows that EMBR-seq is able to detect more genes at different gene expression levels, spanning over three orders of magnitude. Furthermore, we also observed that EMBR-seq derived reads mapped uniformly across the entire length of operons, with modest 3′ and 5′ end bias, suggesting that this method can be used to effectively quantify the expression of genes within operons (Additional file 1: Fig. S5). Finally, we observed that gene expression between the control and mRNA enriched samples were highly correlated (Pearson *r* = 0.97) revealing that the blocking primers do not introduce technical biases in the quantification of gene expression (Fig. 2d). Collectively, these results demonstrate that our new cost-effective method is able to accurately capture the transcriptome of bacterial cells.

### EMBR-seq allows mRNA sequencing from low input total RNA

**Fig. 3:**
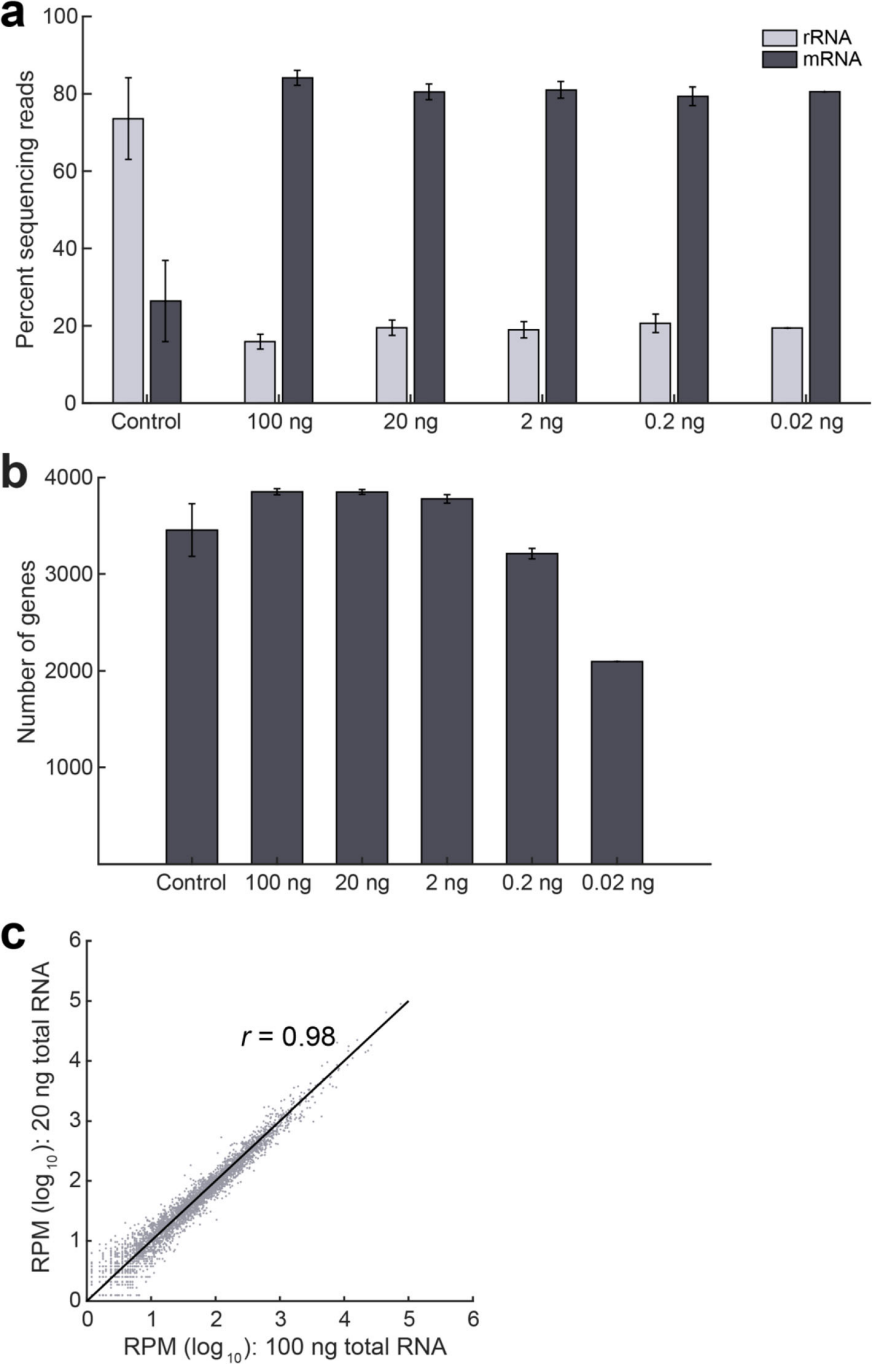
EMBR-seq can quantify the transcriptome from low input total RNA. **a** Similar levels of rRNA depletion and mRNA enrichment are observed when the starting amount of total RNA is decreased from 100 ng to 0.02 ng (*n* ≥ 2, except at 0.02 ng where *n* = 1). The control represents average data of control samples made from different input levels of total RNA. The 100 ng data is reproduced from Fig. 2a. **b** Compared to the control samples, more genes are detected when starting with at least 2 ng input total RNA. Fewer genes are detected when starting total RNA decreases to 0.02 ng. **c** Gene transcript counts are highly correlated (Pearson *r* = 0.98) between 100 ng and 20 ng input total RNA in EMBR-seq. Datasets from lower starting total RNA are also well correlated to the 100 ng samples (Additional file 1: Fig. S6)

In many practical applications involving non-model and non-cultivable bacterial species, the starting amount of total RNA available for RNA sequencing can be limiting. Therefore, we evaluated if we can successfully deplete rRNA and quantify gene expression from lower amounts of input material. We applied EMBR-seq to 20, 2, 0.2 and 0.02 ng of starting total RNA isolated from the exponential growth phase of *E. coli* strain K12. These starting quantities of total RNA were chosen as they are typically below the sensitivity and detection limit of commercial kits and previously reported methods [11, 17]. As before, we observed a greater than 3-fold depletion of rRNA across the range of input starting material, including at the lowest starting amount of 0.02 ng total RNA, with greater than 77% of the reads in the sequencing library deriving from mRNA molecules (Fig. 3a). Similarly, we observed that the total number of genes detected is higher than that in the control samples and is unaffected by the starting input amount of total RNA, except at the lower starting amounts of 0.2 ng and 0.02 ng total RNA (Fig. 3b). Finally, we also observed that gene expression was highly correlated between different amounts of starting total RNA (Fig. 3c and Additional file 1: Fig. S6). These experiments conclusively demonstrate that we can successfully apply EMBR-seq to quantify gene expression from total RNA starting as low as 20 pg.

### rRNA depletion efficiency of EMBR-seq can be further improved through additional blocking primers

**Fig. 4:**
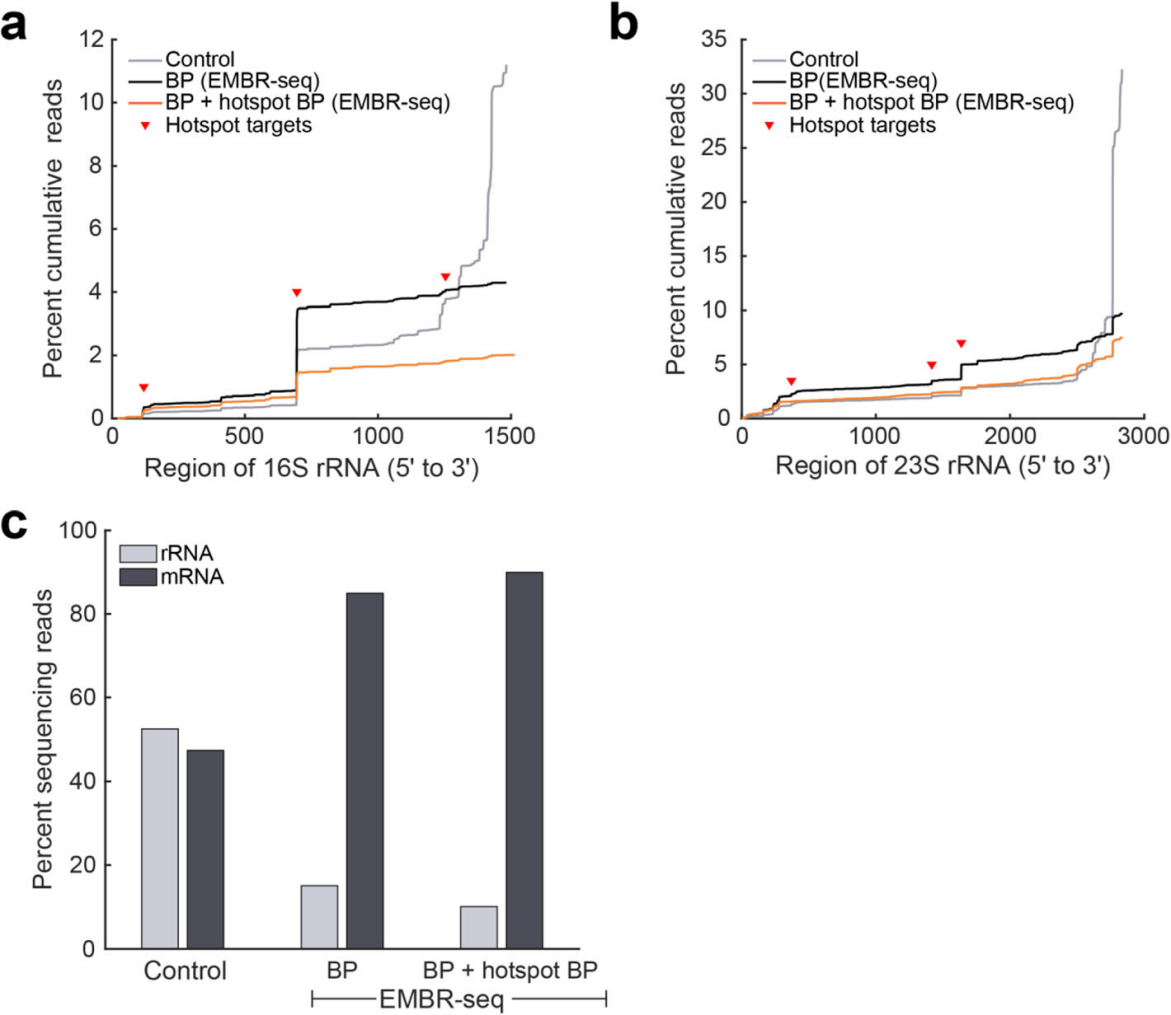
Additional hotspot blocking primers increase the rRNA depletion efficiency of EMBR-seq. **a**, **b** Cumulative percentage of sequencing reads ordered by mapping location along the (**a**) 16S and (**b**) 23S rRNA subunits, from 5′ to 3′ ends of the transcript. In the control group, the majority of mapped reads are derived from the 3′ end together with a few “hotspot” locations (red triangles) along the gene body (gray lines). In EMBR-seq, 3′ end blocking primers sharply reduce the number of reads derived from the 3′ end (black lines) with the remaining rRNA reads primarily deriving from hotspot locations. Additional blocking primers were designed to minimize poly-A tailing and amplification from the vicinity of these coordinates, resulting in further rRNA depletion (orange lines). **c** rRNA depletion and mRNA enrichment is enhanced upon the addition of hotspot blocking primers. With 3′ end and hotspot blocking primers in EMBR-seq, mRNA molecules account for 90% of the mapped reads

In all the EMBR-seq experiments described above, we observed that 13–22% of the mapped reads still derived from rRNA and therefore, we next attempted to further improve the rRNA depletion efficiency of EMBR-seq. Analyzing the mapped coordinates of the rRNA-derived reads showed that while the 3′ blocking primers in EMBR-seq effectively depleted rRNA-derived reads compared to control samples from the 3′ end of rRNA molecules, specific “hotspot” regions along the entire length of the 16S and 23S rRNA were disproportionately abundant in the rRNA capture profile (Fig. 4a, b). We hypothesized that these reads resulted from the combined effects of poly-adenylation of fragmented RNA and biased capture of IVT amplified RNA molecules by random hexamer primers during reverse transcription. This reverse transcription step is part of the final Illumina library preparation protocol where the IVT amplified RNA is first reverse transcribed prior to generation of the Illumina libraries by PCR [20–22]. To minimize reads from these specific rRNA regions, we introduced 3 additional blocking primers per rRNA species that targeted the following hotspot locations: coordinates 107, 682, 1241 on 16S rRNA and coordinates 375, 1421, 1641 on 23S rRNA. We found that these hotspot blocking primers successfully reduced rRNA-derived reads from their target locations in the final sequencing library (Fig. 4a, b). Overall, this resulted in further improvement in the rRNA depletion efficiency of EMBR-seq with only 10% of the mapped reads deriving from rRNA (Fig. 4c). These results demonstrate that EMBR-seq is a versatile technique that can be used to effectively deplete rRNA and can potentially be extended to target and deplete any undesired RNA species.

## Discussion

We have developed a new technology, EMBR-seq, to efficiently deplete rRNA from total RNA, thereby enabling a deeper view of the genome-wide distribution of mRNA in bacterial samples. Sequencing bacterial mRNA poses several challenges; for example, the inability to easily enrich mRNA that typically makes up less than 5% of total RNA and the limiting starting amounts of total RNA that may be available when working with non-cultivable bacterial samples [7–9]. Through the use of a single 3′ blocking primer per rRNA species, EMBR-seq efficiently minimizes the downstream amplification of rRNA molecules, thereby enabling a 4-fold depletion of rRNA in the final sequencing library (Figs. 1 and 2a). In the future, when introducing blocking primers at the 3′ end of an rRNA species, we hypothesize that the rRNA depletion efficiency could be further improved by designing primers with a 5′ overhang or a bulky 5′ modification. As demonstrated in this work, the design of blocking primers at the 3′ end of rRNA molecules efficiently depletes rRNA from high quality total RNA samples; however, certain practical applications can produce degraded and fragmented RNA in which rRNA molecules may be less effectively depleted. While we show that the 3′ blocking primer alone is sufficient to obtain efficient rRNA depletion when starting with degraded *E. coli* total RNA, a more generalized strategy to overcome this challenge in EMBR-seq is to design additional blocking primers per rRNA species, that span the transcript length to minimize amplification of degraded rRNA molecules (Fig. 4 and Additional file 1: Fig. S1).

Starting with total RNA from *E. coli*, we show that efficient depletion of rRNA by EMBR-seq provides higher coverage of the transcriptome at the same sequencing depth (Fig. 2b and Additional file 1: Fig. S4). For example, compared to the control samples, the number of unique genes detected increases from 3628 to 3852 in EMBR-seq (Fig. 2b). In particular, EMBR-seq improves detection of lowly expressed genes below 500 RPM (Fig. 2b). Further, EMBR-seq provides a more in-depth view of the transcriptional landscape without introducing technical artifacts. We find that 99% of the genes detected in the control group are also detected by EMBR-seq, and that gene expression levels between the two groups are highly correlated (Fig. 2c, d).

As EMBR-seq typically uses a single blocking primer per rRNA species, sequence conservation analysis of 16S and 23S rRNA suggests that it is adaptable to other microbial species and complex bacterial communities (Additional file 1: Fig. S7). Recent approaches that employ a large array of probes also achieve a high efficiency of rRNA degradation; however, the need to generate such a large pool of molecules makes it more challenging to extrapolate these methods to evolutionarily distant bacterial species compared to EMBR-seq [16–18]. In addition, the use of just one or a few primers per rRNA species combined with the high level of sample multiplexing reduces cost significantly compared to other methods, enabling cost-effective and high-throughput processing of hundreds of samples simultaneously (Additional file 1: Fig. S3 and Additional file 2). While the cost per rRNA depletion reaction of other reported techniques is an order-of-magnitude higher than EMBR-seq, it is possible that the costs associated with these other methods and commercial kits could be reduced if all reagents and associated steps of the protocol are produced and performed in-house. Finally, beyond depleting rRNA and enriching for mRNA, the approach used in EMBR-seq can potentially also be used to target other high abundance transcripts in total RNA or used to enrich for non-coding RNA in both prokaryotic and eukaryotic systems [28–31].

We also demonstrated that EMBR-seq enables mRNA sequencing of low input RNA samples below the detection limit of commercial kits (Fig. 3a). Bacterial populations frequently contain diverse species, and even isogenic systems have been shown to display substantial cell-to-cell heterogeneity in gene expression that can give rise to dramatic cellular phenotypes [32–37]. Therefore, scaling down bacterial mRNA sequencing techniques to a single-cell level will enable quantification of this variability and provide a better understanding of how transcriptomic heterogeneity regulates cellular function [38, 39]. Over the last few years, a limited number of approaches have been developed to sequence the transcriptome of single bacterial cells. Early proof-of-concept methods were low throughput techniques that sequenced less than 10 single cells and generally suffered from significant technical noise [10, 40, 41]. More recently, Blattman et al. employed combinatorial barcoding to circumvent single cell isolation, enabling high throughput single-cell sequencing of bacterial cell [42]. However, this method did not deplete rRNA, resulting in mRNA detection efficiencies of ~ 2.5–10% (or ~ 200 mRNA per exponential phase *E. coli* cell). As an alternate approach, Imdahl et al. used MATQ-seq to generate sufficient cDNA from individually isolated bacterial cells; however, similar to the previous work, this method also did not deplete rRNA prior to sequencing [43]. In another study, Kuchina et al. combined rRNA depletion with combinatorial barcoding to achieve ~ 5–10% mRNA detection efficiencies in *B. subtilis* [14]. These initial efforts suggest that improved methods could significantly advance single-cell mRNA sequencing in bacteria. EMBR-seq can successfully sequence mRNA from as low as 20 pg of total RNA; therefore, we anticipate that by coupling our rRNA depletion strategy with recent combinatorial barcoding techniques, we will be able to extend EMBR-seq to a single-cell resolution in the future [14, 42].

## Conclusion

EMBR-seq efficiently depletes rRNA and provides a detailed view of the gene expression landscape within bacterial samples. As EMBR-seq depletes rRNA using a single or a few blocking primers per rRNA species, this new method is easily adaptable to other microbes as well as an order-of-magnitude cheaper than other reported techniques and commercial kits that frequently use a large array of probes to remove rRNA from total RNA. Finally, EMBR-seq effectively captures the transcriptome from 500-fold lower starting total RNA compared to commercial kits, thereby providing a powerful new approach to investigate gene expression patterns in rare and non-cultivable bacterial species.

## Methods

### Bacterial strains and culture conditions

*Escherichia coli* MG1655 (ATCC: 700926) overnight cultures were inoculated into fresh LB medium at 1:50 and grown at 37 °C with shaking (150 rpm). Upon reaching the exponential growth phase, the culture was centrifuged at 3000 g for 10 min. The media was removed and the pellet was resuspended in PBS to a concentration of 10^7^ cells per μL. The cells were stored on ice and total RNA extraction was performed immediately.

### RNA extraction

Trizol (Thermo Fisher Scientific, Cat. # 15596018) RNA extraction was performed following the manufacturer’s protocol. Briefly, 10^8^ cells were added to 750 μL Trizol, mixed, and then combined with 150 μL chloroform. After centrifugation, the clear aqueous layer was recovered and precipitated with 375 μL of isopropanol and 0.67 μL of GlycoBlue (Thermo Fisher Scientific, Cat. # AM9515). The pellet was washed twice with 75% ethanol and after the final centrifugation, the resulting pellet was resuspended in RNase-free water.

### EMBR-seq

#### Poly adenylation

100 ng of total RNA in 2 μL was combined with 3 μL poly-A mix, comprised of 1 μL 5x first strand buffer [250 mM Tris-HCl (pH 8.3), 375 mM KCl, 15 mM MgCl_2_, comes with Superscript II reverse transcriptase, Invitrogen Cat. # 18064–014], 1 μL blocking primer mix (see *Primers*), 0.8 μL nuclease-free water, 0.1 μL 10 mM ATP, and 0.1 μL *E. coli* poly-A polymerase (New England Biolabs, Cat. # M0276S). The mixture was incubated at 37 °C for 10 min. In the control group, no blocking primers were added and 1.8 μL of nuclease-free water was added instead. For EMBR-seq with either unmodified or phosphorylated 3′-end blocking primers, the blocking primer mix was prepared by mixing equal volumes of 50 μM blocking primers specific to 5S, 16S and 23S rRNA. For EMBR-seq with hotspot blocking primers, the blocking primer mix was prepared by mixing equal volumes of 100 μM 3′-end blocking primers with 100 μM hotspot blocking primers, such that the final mixture was 50 μM 3′-end primers (3 primers mixed) and 50 μM hotspot primers (6 primers mixed).

#### Reverse transcription

The polyadenylation product was mixed with 0.5 μL 10 mM dNTPs (New England Biolabs, Cat. # N0447L), 1 μL reverse transcription primers (25 ng/μL, see *Primers*), and 1.3 μL blocking primer mix, and heated to 65 °C for 5 min, 58 °C for 1 min, and then quenched on ice. In the control samples, the blocking primers were again replaced with nuclease-free water. Next, 3.2 μL RT mix, consisting of 1.2 μL 5x first strand buffer, 1 μL 0.1 M DTT, 0.5 μL RNaseOUT (Thermo Fisher Scientific, Cat. #10777019), and 0.5 μL Superscript II reverse transcriptase was added to the solution, followed by 1 h incubation at 42 °C. The temperature was then raised to 70 °C for 10 min to heat inactivate Superscript II.

#### Second strand synthesis

49 μL of the second strand mix, containing 33.5 μL water, 12 μL 5x second strand buffer [100 mM Tris-HCl (pH 6.9), 23 mM MgCl_2_, 450 mM KCl, 0.75 mM β-NAD, 50 mM (NH_4_)_2_ SO_4,_ Invitrogen, Cat. # 10812–014], 1.2 μL 10 mM dNTPs, 0.4 μL *E. coli* ligase (Invitrogen, Cat. # 18052–019), 1.5 μL DNA polymerase I (Invitrogen, Cat. # 18010–025), and 0.4 μL RNase H (Invitrogen, Cat. # 18021–071), was added to the product from the previous step. The mixture was incubated at 16 °C for 2 h. cDNA was purified with 1x AMPure XP DNA beads (Beckman Coulter, Cat. # A63881) and eluted in 24 μL nuclease-free water that was subsequently concentrated to 6.4 μL.

#### In vitro transcription

The concentrated solution was mixed with 9.6 μL of Ambion in vitro transcription mix (1.6 μL of each ribonucleotide, 1.6 μL 10x T7 reaction buffer, 1.6 μL T7 enzyme mix, MEGAscript T7 Transcription Kit, Thermo Fisher Scientific, Cat. # AMB13345) and incubated at 37 °C for 13 h. Next, the aRNA was treated with 6 μL EXO-SAP (ExoSAP-IT™ PCR Product Cleanup Reagent, Thermo Fisher Scientific, Cat. # 78200.200.UL) at 37 °C for 15 min followed by fragmentation with 5.5 μL fragmentation buffer (200 mM Tris-acetate (pH 8.1), 500 mM KOAc, 150 mM MgOAc) at 94 °C for 3 min. The reaction was then quenched with 2.75 μL stop buffer (0.5 M EDTA) on ice. The fragmented aRNA was size selected with 0.8x AMPure RNA beads (RNAClean XP Kit, Beckman Coulter, Cat. # A63987) and eluted in 15 μL nuclease-free water. Thereafter, Illumina libraries were prepared as described previously [20].

### EMBR-seq with TEX digestion

To test the Terminator™ 5′-phosphate-dependent exonuclease (Lucigen, Cat. # TER5120), 100 ng of total RNA in 2 μL was combined with 18 μL TEX mix, comprised of 14.5 μL nuclease free water, 2 μL Terminator 10x buffer A, 0.5 μL RNAseOUT, and 1 μL TEX. The solution was incubated at 30 °C for 1 h and quenched with 1 μL of 100 mM EDTA. The product was purified with 1x AMPure RNA beads and eluted in 10 μL nuclease-free water and concentrated to 2 μL. This TEX digested total RNA was then used as starting RNA in the EMBR-seq protocol described above.

### EMBR-seq bioinformatic analysis

Paired-end sequencing of the EMBR-seq libraries was performed on an Illumina NextSeq 500. All sequencing data has been deposited to Gene Expression Omnibus under the accession number GSE149666. In the sequencing libraries, the left mate contains information about the sample barcode (see *Primers*). The right mate is mapped to the bacterial transcriptome. Prior to mapping, only reads containing valid sample barcodes were retained. Subsequently, the reads were mapped to the reference transcriptome (*E. coli* K12 substr. MG1655 cds ASM584v2) using Burrows-Wheeler Aligner (BWA) with default parameters.

### Analysis of detection bias in EMBR-seq

*E. coli* operons were downloaded from RegulonDB [44]. Operons with at least 2 genes were included for this analysis. The data from EMBR-seq libraries with 100 ng starting material was mapped to *E. coli* K12 substr. MG1655 reference genome (ASM584v2). For each read that maps within an operon, the distance of the mapped location from the 3′ end of the operon was calculated, accounting for the read length. Next, the operons were discretized into 50 bins, and all operons with more than 200 unique reads were considered for downstream analysis. The number of reads in each bin was then normalized by the total number of reads in each operon, and the average of the relative reads within each bin was calculated. To compare bacterial data from EMBR-seq to mammalian data from CEL-seq, we downloaded CEL-seq data reported in Grün et al. (GEO Accession: GSM1322290) and performed similar analysis for the mouse genes [45].

### Sequence conservation of 16S and 23S rRNA

16S rRNA sequences from 4000 species were obtained from *rrnDB* [46], while 23S rRNA sequences from 119 species were selected from NCBI RefSeq [47]. Next, the last 100 bases from the 3’end of each sequence were aligned using Clustal Omega [48]. Shannon entropy for each aligned base location was then calculated such that the maximal entropy value was 1. Five possibilities were allowed: “A”, “T”, “C”, “G”, and “-”.

### Primers

Reverse transcription primers are shown below with the 6-nucleotide sample barcodes underlined [20]:

GCCGGTAATACGACTCACTATAGGGAGTTCTACAGTCCGACGATCNNNNNN(NNNNNN)TTTTTTTTTTTTTTTTTTTTTTTTV

The following five barcodes were used in this study:

AGACTC

AGCTTC

CATGAG

CAGATC

TCACAG

Blocking primers:

5S 5′-ATGCCTGGCAGTTCCCTACTCTCGCATGGG-3′

16S 5′-TAAGGAGGTGATCCAACCGCAGGTTCCCCT-3′

23S 5′-AAGGTTAAGCCTCACGGTTCATTAGTACCG-3′

In the case of the 3′ phosphorylated primers, all blocking primers have a 3′ phosphorylation modification.

Hotspot blocking primers:

16S primer for hotspot at position 107:

5′-GGCACATCCGATGGCAAGAGGCCCGAAGGT-3′.

16S primer for hotspot at position 682:

5′-TCCTGTTTGCTCCCCACGCTTTCGCACCTG-3′.

16S primer for hotspot at position 1241:

5′-CCGTGGCATTCTGATCCACGATTACTAGCGATTCCG-3.

23S primer for hotspot at position 375:

5′-CGCCTTTCCCTCACGGTACTGGTTCACTATCGG-3′.

23S primer for hotspot at position 1421:

5′-TTGCTTCAGCACCGTAGTGCCTCGTCATCA-3′.

23S primer for hotspot at position 1641:

5′-GCAGCCAGCTGGTATCTTCGACTGATTTCAGC-3′.

Each primer is designed to anneal approximately 100 bp downstream of the hotspot. The exact position and length of each primer was adjusted to ensure the T_m_ was above 65 °C.

## Supplementary information

**Additional file 1 Figure S1.** EMBR-seq effectively depletes rRNA from fragmented total RNA. **Figure S2.** Combining TerminatorTM 5′-phosphate-dependent exonuclease (TEX) digestion with EMBR-seq does not improve rRNA depletion. **Figure S3.** Cost associated with performing EMBR-seq. **Figure S4.** Higher number of genes detected in EMBR-seq is not dependent on the sequencing depth. **Figure S5.** Distribution of reads along *E. coli* operons in EMBR-seq. **Figure S6.** Gene transcript count correlation between different input total RNA amounts in EMBR-seq. **Figure S7.** Quantification of 16S and 23S rRNA sequence conservation using Shannon entropy. **Table S1.** Comparison of rRNA depletion methods.

**Additional file 2.**

## Data Availability

The datasets generated and analyzed in the current study have been deposited to GEO under the accession number GSE149666.

## Abbreviations

IVT: In vitro transcription
LB: Lysogeny broth
RNase H: Ribonuclease H
TEX: Terminator™ exonuclease
