# Supplementary material for: Efficient and cost-effective bacterial mRNA sequencing from low input samples through ribosomal RNA depletion
Source: BMC Genomics. 2020 Oct 16;21:717. doi: 10.1186/s12864-020-07134-4 (PMC7565789; doi:10.1186/s12864-020-07134-4)
Supplement: Supplementary file 1 — Additional file 1 Figure S1. EMBR-seq effectively depletes rRNA from fragmented total RNA. Figure S2. Combining TerminatorTM 5′-phosphate-dependent exonuclease (TEX) digestion with EMBR-seq does not improve rRNA depletion. Figure S3. Cost associated with performing EMBR-seq. Figure S4. Higher number of genes detected in EMBR-seq is not dependent on the sequencing depth. Figure S5. Distribution of reads along E. coli operons in EMBR-seq. Figure S6. Gene transcript count correlation between different input total RNA amounts in EMBR-seq. Figure S7. Quantification of 16S and 23S rRNA sequence conservation using Shannon entropy. Table S1. Comparison of rRNA depletion methods. [file 12864_2020_7134_MOESM1_ESM.pdf]

Supplementary Information

Efficient and cost-effective bacterial mRNA sequencing from low input samples through ribosomal RNA depletion

Chatarin Wangsanuwat[1,2,†], Kellie A. Heom[1,2,†], Estella Liu[1], Michelle A. O'Malley[1,2], and Siddharth S. Dey[1,2,3,*]

[1] Department of Chemical Engineering, University of California Santa Barbara, Santa Barbara, CA 93106, USA.

[2] Center for Bioengineering, University of California Santa Barbara, Santa Barbara, CA 93106, USA.

[3] Neuroscience Research Institute, University of California Santa Barbara, Santa Barbara, CA 93106, USA.

† These authors contributed equally to this work.

*Correspondence should be addressed to S.S.D (sdey@ucsb.edu).

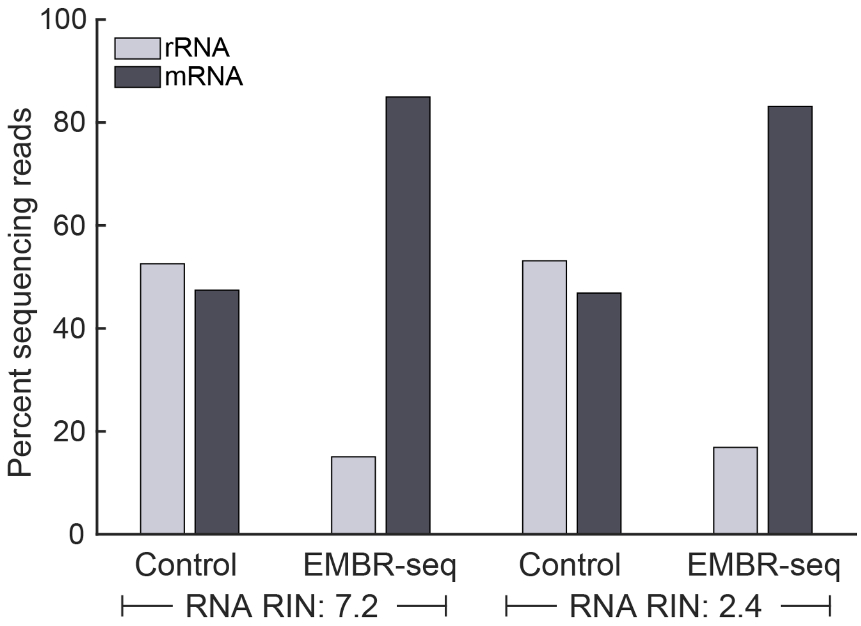

Figure S1: EMBR-seq effectively depletes rRNA from fragmented total RNA. EMBR-seq depletes rRNA to 15% and 17% of the mapped reads for total RNA samples with RIN scores of 7.2 and 2.4, respectively. In both cases, mRNA accounts for more than 80% of the mapped reads. These experiments were performed starting with 100 ng total RNA from *E. coli.*

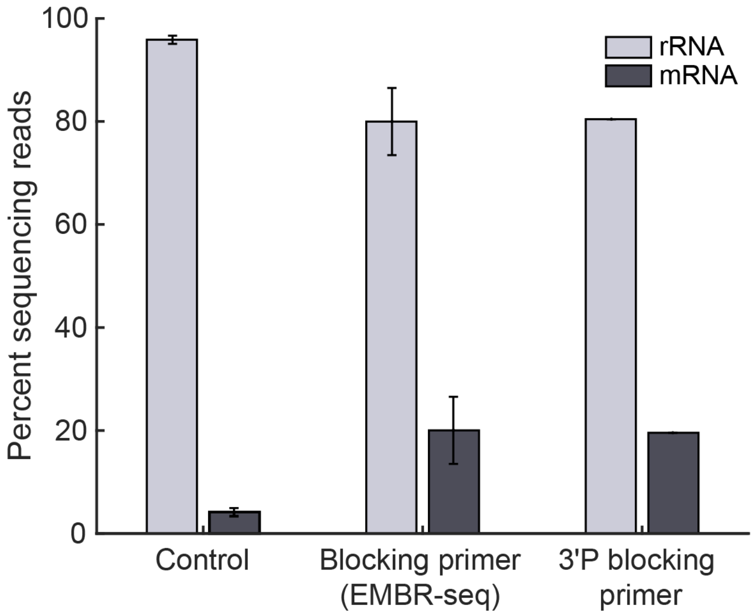

Figure S2: Combining Terminator™ 5'-phosphate-dependent exonuclease (TEX) digestion with EMBR-seq does not improve rRNA depletion. Performing TEX digestion prior to EMBR-seq results in less efficient rRNA depletion and mRNA enrichment compared to experiments without TEX (Fig. 2a) ($n \geq 2$, except in TEX + 3'P blocking primer where $n = 1$). These experiments were performed starting with 100 ng total RNA from *E. coli.* Error bars represent standard deviations.

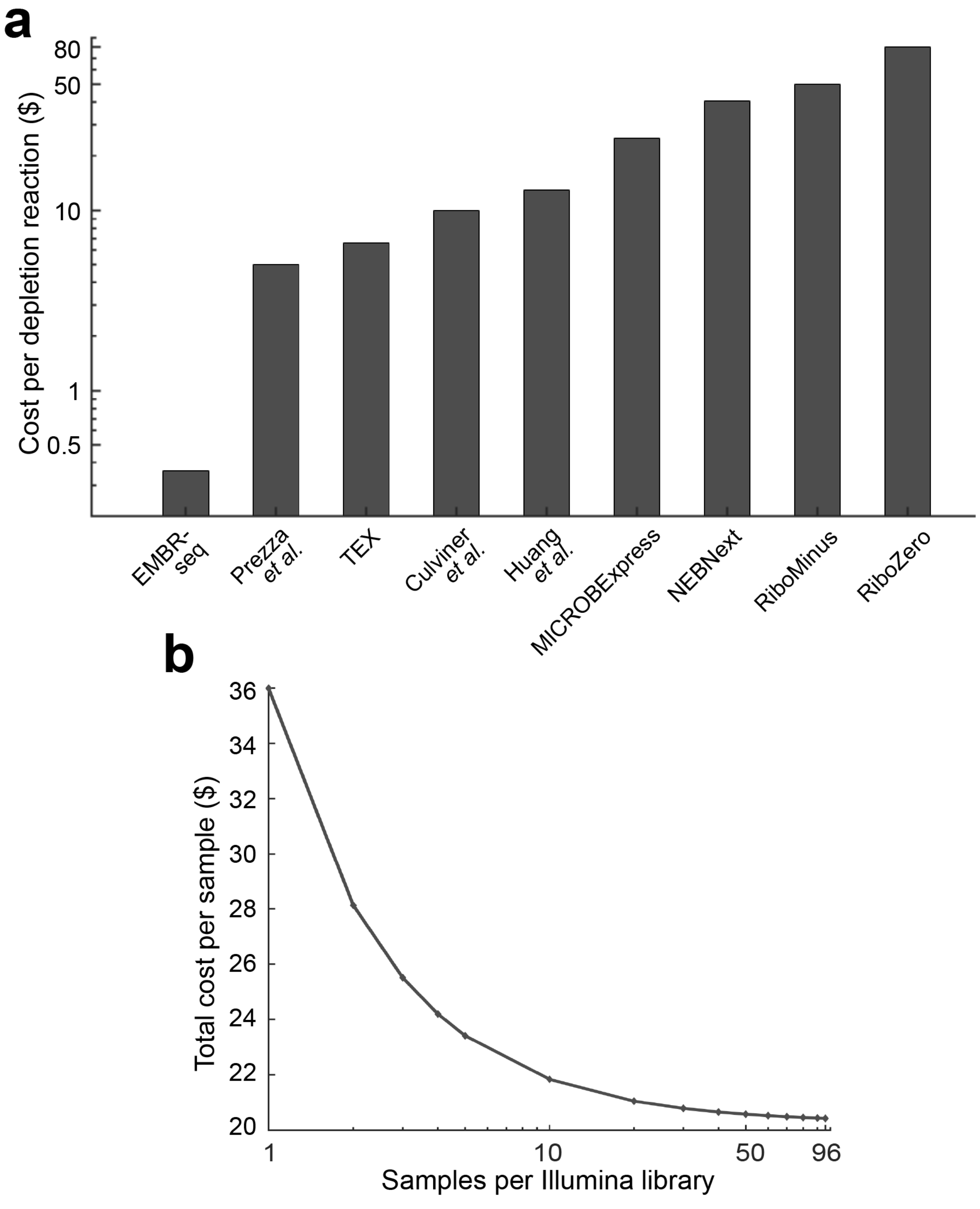

Figure S3: Cost associated with performing EMBR-seq. (**a**) The cost of performing rRNA depletion in EMBR-seq is ~$0.40 per reaction. The cost per reaction in EMBR-seq is an order of magnitude lower than other published rRNA depletion methods and commercial kits (Additional

file 1: Table S1 and Additional file 2). (**b**) The plot shows the total cost for the complete EMBR-seq protocol per sample (starting from total bacterial RNA extraction to Illumina library preparation) as a function of the number of samples multiplexed (using the sample barcodes in the RT primer) per Illumina library. Starting from 1 sample per Illumina library to 96 samples per Illumina library, the total cost drops from $36 to $20 per sample.

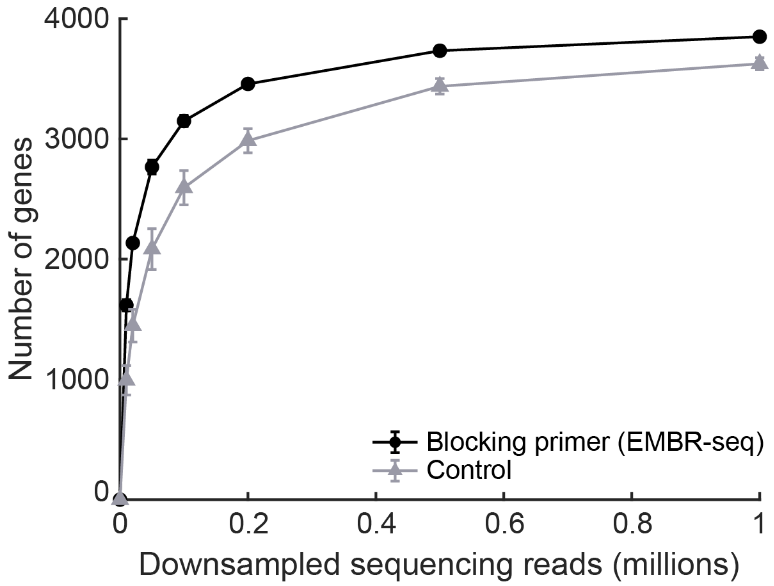

Figure S4: Higher number of genes detected in EMBR-seq is not dependent on the sequencing depth. To ensure that the number of genes detected in EMBR-seq samples compared to the control samples is not an artifact of sequencing depth, we downsampled the mapped sequencing reads to show that EMBR-seq detects more genes at different levels of downsampling. The figure also shows that the number of genes detected does not increase substantially beyond ~0.5 million mapped reads, suggesting that our sequencing libraries have been sequenced at sufficient depth ($n = 3$). Error bars represent standard deviations. For the EMBR-seq group, error bars are of the same scale as the size of the data points.

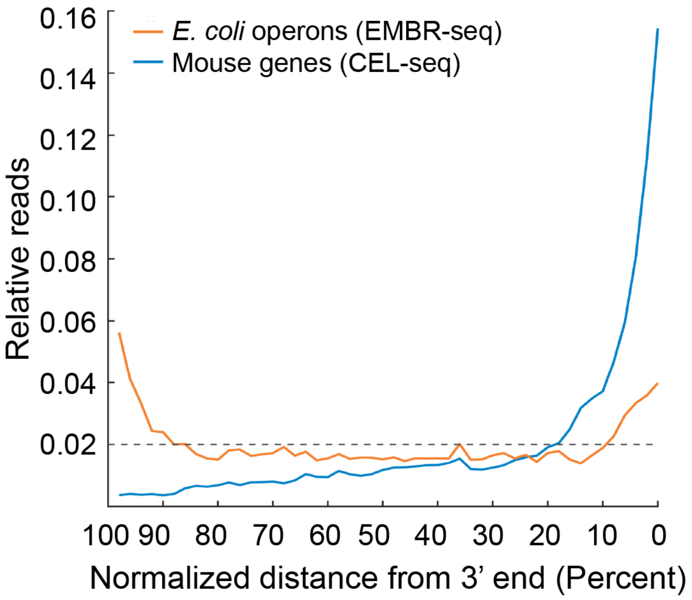

Figure S5: Distribution of reads along *E. coli* operons in EMBR-seq. The panel shows the distribution of reads along *E. coli* operons obtained from EMBR-seq and mouse genes obtained from CEL-seq. The normalized distance from the 3' end is based on discretizing the *E. coli* operons and mouse genes into 50 bins. The dotted line indicates the expected distribution of reads from each bin in the absence of any detection bias. The *E. coli* data is obtained from 100 ng starting total RNA.

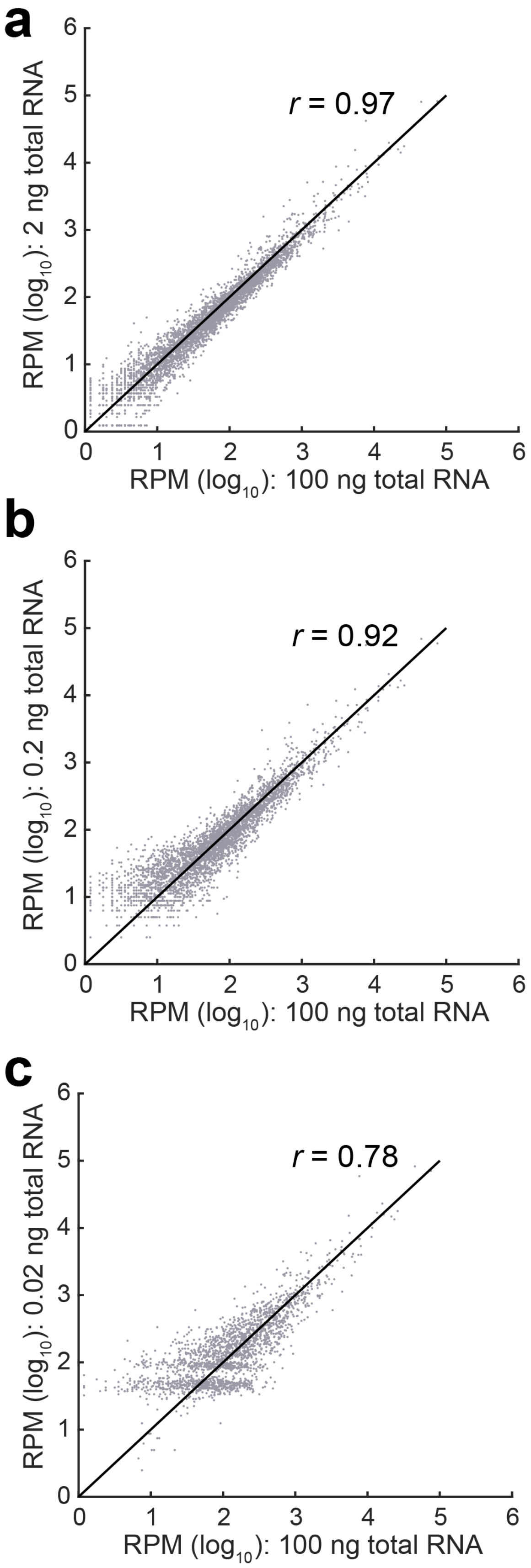
a
r = 0.97
RPM (log10): 2 ng total RNA
RPM (log10): 100 ng total RNA
b
r = 0.92
RPM (log10): 0.2 ng total RNA
RPM (log10): 100 ng total RNA
c
r = 0.78
RPM (log10): 0.02 ng total RNA
RPM (log10): 100 ng total RNA
0
1
2
3
4
5
6

Figure S6: Gene transcript count correlation between different input total RNA amounts in EMBR-seq. (**a-c**) Panels show gene transcript count Pearson correlations between 100 ng starting total RNA and lower input total RNA in EMBR-seq. As expected, the Pearson correlation drops when starting with lower amounts of total RNA. These experiments were performed with total RNA from *E. coli.*

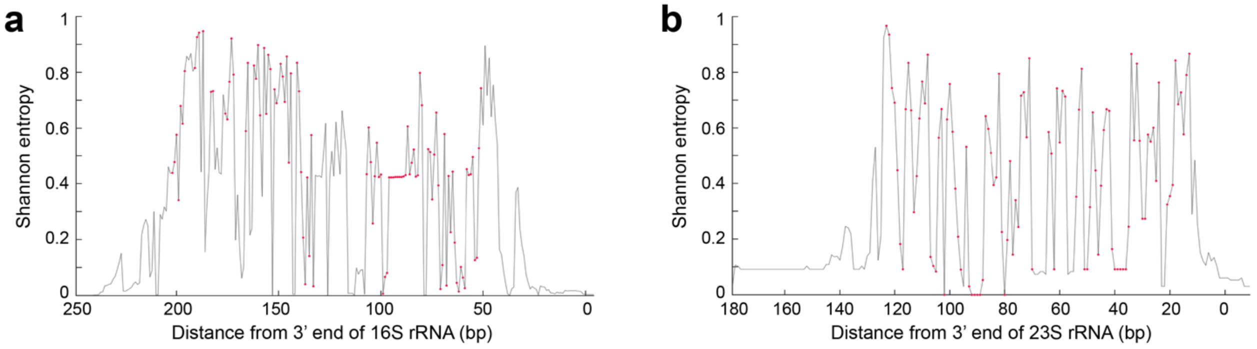

Figure S7: Quantification of 16S and 23S rRNA sequence conservation using Shannon entropy. (**a**,**b**) The panels show Shannon entropy scores for the sequence alignment of the last 100 bases of 16S and 23S rRNA from 4000 and 119 species, respectively. The red dots are the locations of the *E. coli* bases. The minimum entropy score of zero indicates that a position is completely conserved across all species analyzed, and the maximum Shannon entropy of 1.0 indicates that the bases at a location are uniformly distributed among species, or minimally conserved.

Table S1: Comparison of rRNA depletion methods.

Supplier or publication	Kit or method name	Working principle	Cost/sample ($, approx.)	Minimal input	Percent rRNA left	Advantages	Disadvantages
This work	EMBR-seq	Poly-A tailing with rRNA blocking primers	0.36	20 pg	~10-22%	1. Inexpensive and small up-front cost<br>2. Simple design: 1 oligo per type of rRNA<br>3. Shown to work for starting material as low as 20 pg	1. Does not deplete as much rRNA as other methods<br>2. Minor detection bias
Prezza et al., 2020 [1]	DASH	Cas9-mediated cleavage of rRNA-derived cDNA	3-7	~0.4 ng	~10-50%	1. Shown to work for both gram-negative bacteria and anaerobic gut bacteria<br>2. Low cost<br>3. Has software for automated guide RNA design<br>4. Shown to work for starting material as low as 400 pg<br>5. Does not lower the amount of starting material for initial cDNA synthesis and PCR amplification	1. Requires more than 100 primer sequences, need around 650-800 oligos for more efficient depletion<br>2. Does not deplete as much rRNA as other methods<br>3. Depletion efficiency is variable and depends on the concentration ratio of sgRNA and Cas9 to the cDNA, which might require some optimization
Lucigen	Terminator exonuclease (TEX)	5' selective rRNA degradation	6.6	1 ug (recommended by Lucigen);<br>100 ng (this work);<br>single bacteria cells [2]	~85-90% [3]	1. Simple enzymatic reaction that requires little design effort and is straightforward to perform in the lab	1. Poor rRNA depletion<br>2. Appears to worsen mRNA detection in combination with other techniques<br>3. Might introduce 5' detection bias

Supplier or publication	Kit or method name	Working principle	Cost/sample ($, approx.)	Minimal input	Percent rRNA left	Advantages	Disadvantages
Culviner et al., 2020 [4]	\	Oligo-based rRNA pull-down	10	2 ug	~20-25%	1. Has an algorithm for designing oligos for any species or combination of species of interest<br>2. Shown to work for at least 3 common bacterial strains	1. Requires multiple oligo optimization cycles *in silico*, up to 100 rounds<br>2. New optimization step might be required for every new combination of species<br>3. Up-front cost for the oligos<br>4. Only microgram-level starting material reported
Huang et al., 2019 [5]	\	RNase H-based	12.94	100 ng	<5% to ~25%	1. Shown to work for bacteria from 3 distinct phyla<br>2. Oligo probes can be applied to closely related species<br>3. Very good rRNA depletion<br>4. Has a simple tool to design probe libraries<br>5. Option to order probes or synthesize in house	1. Requires > 80 oligos<br>2. Up-front cost for the oligos<br>3. Lowest input reported is 100 ng
Thermo Fisher Scientific	MICROBExpress	Oligo-based rRNA pull-down	25	2 ug	1 to <10%	1. Excellent rRNA depletion<br>2. Works for many different gram-positive and gram-negative bacterial species	1. Cheaper alternate oligo-based pull-down methods available<br>2. Likely not easily customizable to other species not validated by the manufacturer<br>3. Only microgram-level starting material reported

Supplier or publication	Kit or method name	Working principle	Cost/sample ($, approx.)	Minimal input	Percent rRNA left	Advantages	Disadvantages
NEB	NEBNext rRNA depletion kit (bacteria)	RNase H-based	40.5	10 ng	<2%	1. Excellent rRNA depletion<br>2. Compatible with both gram-positive and gram-negative organisms, shown to work well across at least 20 different bacterial species	1. Cheaper alternate RNase H-based methods available<br>2. Likely not easily customizable to other species not validated by the manufacturer
Thermo Fisher Scientific	RiboMinus Transcriptome Isolation Kit, bacteria	Oligo-based rRNA pull-down	50	2 ug	<2%	1. Excellent rRNA depletion	1. Cheaper alternate oligo-based pull-down methods available<br>2. Likely not easily customizable to other species not validated by the manufacturer<br>3. Only microgram-level starting material reported
Illumina	RiboZero Plus rRNA depletion kit	RNase H-based	80 (reported by Prezza et al, 2020 [1])	10 ng	<2%	1. Excellent rRNA depletion<br>2. Shown to work well across at least 25 different species<br>3. Works for both prokaryotic and eukaryotic samples	1.Cheaper alternate RNase H-based methods available<br>2. Likely not easily customizable to other species not validated by the manufacturer

References

1. Prezza G, Heckel T, Dietrich S, Homberger C, Westermann AJ, Vogel J. Improved bacterial RNA-seq by Cas9-based depletion of ribosomal RNA reads. RNA. 2020;26:1069–78.

2. Kuchina A, Brettner LM, Paleologu L, Roco CM, Rosenberg AB, Carignano A, et al. Microbial single-cell RNA sequencing by split-pool barcoding. preprint. bioRxiv; 2019. doi:10.1101/869248.

3. He S, Wurtzel O, Singh K, Froula JL, Yilmaz S, Tringe SG, et al. Validation of two ribosomal RNA removal methods for microbial metatranscriptomics. Nat Methods. 2010;7:807–12.

4. Culviner PH, Guegler CK, Laub MT. A Simple, Cost-Effective, and Robust Method for rRNA Depletion in RNA-Sequencing Studies. mBio. 2020;11:e00010-20.

5. Huang Y, Sheth RU, Kaufman A, Wang HH. Scalable and cost-effective ribonuclease-based rRNA depletion for transcriptomics. Nucleic Acids Res. 2020;48:e20.
